# Exploring the mechanism through which a child-friendly storybook addresses barriers to child-participation during HIV care in primary healthcare settings in KwaZulu-Natal, South Africa

**DOI:** 10.1186/s12889-021-10483-8

**Published:** 2021-03-16

**Authors:** Chipo Mutambo, Kemist Shumba, Khumbulani W. Hlongwana

**Affiliations:** 1 The Discipline of Public Health Medicine, School of Nursing and Public Health, University of KwaZulu-Natal, Durban, South Africa; 2 The Discipline of Psychology, School of Applied Human Sciences, University of KwaZulu-Natal, Durban, South Africa

**Keywords:** KidzAlive, Talk tool storybook, Child-participation, Storytelling, Child-centred care, Theory of change

## Abstract

**Background:**

Healthcare workers (HCWs) in South Africa widely use job-aids as practical tools to enhance the provision of HIV services, thereby improving patient-provider interactions during the care process. Job-aids are visual support materials that provide appropriate information using graphics and words in a simple and yet effective manner. We explored the mechanism through the KidzAlive Talk tool storybook (Talk tool), a child-centred job-aid for HCWs that facilitates child-participation during HIV consultations in primary healthcare (PHC) clinics implementing the KidzAlive model.

**Methods:**

The study was conducted in PHC clinics across four districts; namely: uMkhanyakude, Zululand, uMgungundlovu, and eThekwini in KwaZulu-Natal (KZN), South Africa. We conducted in-depth interviews with children (*n* = 30), their primary caregivers (PCGs) (n = 30), and KidzAlive trained and mentored HCWs (*n* = 20). Data were collected in both English and isiZulu languages through user-specific, structured in-depth interviews. All the interviews were audio-recorded (with participants’ assent and consent, respectively). Data were transcribed verbatim, prior to translating the isiZulu transcripts to English. Translations were done by a member of the research team competent in both languages. Electronic data were imported to NVivo 10 for analysis and subsequently analysed using a thematic analysis method followed by a constant comparative and modified grounded theory analysis method.

**Results:**

The findings identified the following barriers to child-participation: Primary caregiver limiting the child’s involvement due to fear of traumatising them; HCWs’ limited knowledge and skills to deliver child-centred HIV care; childhood developmental stage-related limitations and healthcare institutional paternalism. The Talk tool addresses the above barriers by using simple language and terminology to cater for children at various stages of development; alleviating HCWs’ and PCGs’ fear of possible psychological harm to the child; using storytelling and colourful cartoon illustrations for child edutainment; Being versatile by allowing for multiple utility and tackling institutional paternalism that limit child-involvement in the process of care.

**Conclusions:**

This study provided evidence on how the Talk tool storybook addresses barriers to child-participation in the HIV care process. The evidence generated from this study is compelling enough to recommend the scale-up of this innovation in low-resource settings.

**Supplementary Information:**

The online version contains supplementary material available at 10.1186/s12889-021-10483-8.

## Background

Participation of children living with HIV (CLWHIV) in healthcare is increasingly gaining traction in primary healthcare clinics (PHCs) in resource-poor settings given the chronic nature of HIV. Further, it results in children frequenting these facilities for their repeat prescriptions and care [1–6]. The realisation by healthcare providers in resource-constrained settings that scale-up of antiretroviral drugs requires people living with HIV (PLWHIV), including children, to adhere to medical advice and medication has been one of the important considerations [7–10]. For PLWHIV to cooperate, they need to be active participants during their care journey [11, 12]. Children living with HIV can no longer be passive bystanders during their care and care consultation [13]. The increased participation of children on chronic medication due to the proliferation of child-centred approaches has shown evidence of a positive impact on children’s experiences of optimal care, satisfaction, and positive health outcomes in resource-rich countries [2, 3, 5]. Therefore, poor resource settings are also beginning to adopt these important lessons by advocating for increased child-participation in response to the increasing number of CLWHIV on antiretroviral therapy (ART) who now, due to the scale-up of the Universal Treat All policy, need quality care in PHCs [13, 14].

The concept of “participation” has different definitions in different fields from sociology, politics to healthcare [15]. This study adopted the definition of “child participation” suggested by Harry Shier [16] who identifies five levels of participation by children in healthcare decisions as follows: children must be listened to; children are facilitated in expressing their views; children’s views are considered; children must be involved in decision-making processes, and children must share power and responsibility for decision-making. However, other studies in HIV care synonymously use terminologies including “involvement”, “collaboration” and “partnership” of patients, “client”, “consumer”, and “user” participation in healthcare and “patient-centred care” to mean “participation” [15].

Despite the existence of increasing evidence on the benefits of patient involvement in HIV care processes, the participation of CLWHIV in healthcare provision in resource-constrained settings remains problematic as often these children remain passive bystanders during their own HIV care [3, 4]. Exclusion of children during the care process has been widely reported to result in anxiety, confusion, and anger, culminating in increased missed appointments and non-adherence to medical advice or medication [5, 17–20]. Conversely, the early participation of CLWHIV has largely been associated with increased ease of status disclosure, increased adherence to medication and improved health outcomes [21, 22]. Consultation and sharing of information about the child’s HIV positive serostatus with the child (HIV status disclosure) in the presence of the HCW has proven effective to forging partnerships between the parties involved as it promotes transparency, truthfulness, and trust [21, 23–25].

However, non-disclosure to CLWHIV remains the key barrier to child-participation during care consultations with HCWs in South Africa. Generally, disclosure rates among children aged 0–14 in low resource settings, including South Africa are as low as less than 8% [19, 20, 26, 27]. A study conducted in rural KwaZulu-Natal reported that 73% of children aged 5–18 years were unaware of their HIV seropositive status and were at increased risk of non-adherence [28]. Studies in South Africa attribute delayed disclosure to HIV positive children to PCG fear of emotional harm and being blamed by their children, families, and community members [18–20]. PCGs of HIV positive children often struggle with whether to disclose the HIV status, including when, and how to talk to children under their care about their HIV seropositive status [27]. Several studies have reported that some PCGs do not disclose to their children simply because they do not know how to do so [29]. Similarly, HCWs often avoid engaging with HIV positive children, fearing that they may inadvertently disclose to children that are undisclosed to; and may be using age-inappropriate language, words, approach, or techniques [27, 29]. Stigma is one of the main drivers of non-disclosure in South Africa, as both HCWs and PCGs avoid discussions regarding a transmissible, life-threatening, highly stigmatised, and incurable infection with children [30]. In addition, some studies have found that HCWs prefer to only discuss the child’s illness with their PCG, as they perceive children to be incapable of either coherently communicating during healthcare consultations or meaningfully contribute to the decision-making processes regarding their care [31–33].

The challenges discussed above are exacerbated by the insufficient numbers of HCWs in resource-limited settings [3]. Limited training in child-centred HIV counselling and disclosure support remains pervasive in these settings [3, 27, 29, 34]. Studies conducted in South Africa have reported that HCWs providing HIV care to children are overwhelmed, undertrained and frequently have little to no knowledge of appropriate methods of HIV disclosure [20, 35, 36]. They are also constrained in terms of culturally appropriate resources to help support caregivers during the process of HIV disclosure to children and adolescents [34, 37, 38]. In such settings, cost-effective and practical tools for improving HCW capacity to remember HIV counselling, testing and disclosure guidelines are necessary to alleviate some of these challenges [23, 37–39]. In addition, the use of multi-purpose simplified guidelines a potential to capacitate HCWs with knowledge and guidance to deliver child-centred HIV care to children and psychosocial support to their PCGs [13, 38].

Job-aids have proved to be effective in enhancing child-HCW-PCG interactions during HIV care [3, 21, 40]. They enhance HCWs’ memory, reduce guesswork and ensure adherence to healthcare guidelines and reduce costs associated with training and retraining. Furthermore, job-aids ensure that HCWs have consistent access to accurate and simplified information [21, 41]. Job-aids are defined as “an external device or cognitive artefact that provides just-in-time knowledge and information to help individuals with tasks by directing, guiding, and enhancing performance” [40, 42]. Examples of job-aids that can be used in the process of providing care to children, include checklists, algorithms (simplified flowcharts), puppets [43], storybooks [44], comic books, dolls, games and digital media [3, 40, 45], leveraged during healthcare provision to increase HCW-child-PCG engagement, reduce children’s anxiety, and increase understanding of the child’s illness and its management [3]. These job-aids have proven to be beneficial in providing health education and improving healthcare knowledge and clinical outcomes in both resource-rich and resource-limited settings [40, 42, 46, 47]. Other studies have also applauded job-aids for being financially feasible and reducing marginal costs to the already overstretched healthcare budgets when compared to other resource-extensive HCW capacity building interventions [42, 46].

There is an increased focus on adopting child-focussed counselling approaches such as play-therapy when providing care to CLWHIV. Play therapy techniques allow counsellors to access the inner world of a child, connect and assist the child make sense of real world problems and discover solutions [48]. Play therapy encourages the development of a therapeutic relationship between a counsellor and a child. This relationship enables the child’s play to become a source of information, which the counsellor observes to gain insight into the inner world of the child and it allows the child to freely express underlying emotions and to try out new ways of thinking and behaving [48, 49]. By adopting storytelling as a play therapy technique, the counsellor uses metaphors which are considered the “language” of play. This allows the child to protect the self by distancing themselves from painful themes and dealing with them symbolically [48, 50]. While the use of play therapy techniques is well established and recognised in the more developed world, its proliferation in resource-limited countries in Africa remains limited [13]. This is probably due to lack of affordability and inadequate number of trained service providers, such as psychologists and psychiatrists, which limit access to quality healthcare by most vulnerable children hailing from poor economic backgrounds.

It is, therefore, reasonable to investigate the potential contribution of a cost-effective play therapy-based techniques in sub-Saharan Africa, implemented through capacitating HCWs already tasked to provide curative and therapeutic services to CLWHIV in PHCs. These HCWs would need to receive training on the utilisation of these play therapy-based counselling techniques in the context of HIV, and the African interpretation of play. Innovators working in the HIV sector have already developed some ingenious play-based job-aids aimed at offering guidance to HCWs providing care to HIV infected children in primary care settings [3, 21, 47]. These job-aids were designed to capacitate the HCW with knowledge on how to craft age-appropriate and child-friendly messages through storytelling to keep children engaged [3, 21, 33], thereby enhancing patient-provider communication. A good example of play-based job-aids that have been used in sub-Saharan Africa recently includes the disclosure cartoon book, entitled “Why I take my medicine,” which was developed in Namibia as part of HIV care and support for children. This cartoon book uses pictures and child-friendly terminology to improve the disclosure process for children and promote their adherence to medication and improve health outcomes [3, 21, 39]. In this storybook, incorporating storytelling into HIV psychosocial interventions for children using animal characters befits the South African context where stories are traditionally used to educate and entertain children [51–54].

Similarly, the KidzAlive Talk tool storybook (Talk tool) [55], which is the topic of this study is also a play and cartoon-based job-aid that was developed to assist HCWs providing care and support to HIV seropositive children receiving HIV care and support in PHCs in South Africa. The Talk tool is a colourful, animated storybook starring interesting human and animal characters. The main characters of the story are “Sibusiso the Frog”, his grandfather “Mkhulu Noah”, and “Nurse Thelma”. In the story, Sibusiso learns or acts out the process of receiving HIV care at a PHC facility where he is taken by his grandfather and is provided care by the nurse. Both “Mkhulu Noah” and “Nurse Thelma” provide the necessary social support structure to Sibusiso in managing his HIV status. This story uses language deemed suitable for enhancing children’s comprehension of HIV related information such as referring to medication as “Goodnight medicine that put germs to sleep”, HIV as a “Germ in your body” and CD4 cells as “Soldiers that fight the germs” [38].

Anecdotal information suggests that the Talk tool is an efficacious approach to the provision of HIV care to children [38, 56]. However, a formal evaluation of HCWs, children and PCGs’ perceptions of the Talk tool and its effect on improving child participation during HIV service consultations has not been conducted. Therefore, this study attempts to partly close this gap by exploring users’ experiences and perceptions of the Talk tool in a bid to understand how it contributes to mitigating barriers to child engagement during HIV care.

### Overview of the KidzAlive intervention

KidzAlive was conceptualised and illustrated by Zoë-Life in 2006 as a response to the challenges faced by HCWs when providing HIV care to children, and the limited involvement and participation of children in the care process. Zoë-Life, a South African based non-governmental organisation (NGO) has been a technical support partner to South Africa’s National Department of Health (NDoH) in child and adolescent HIV differentiated care for more than 12 years. Zoë-Life continues to support the NDOH by developing innovative interventions for supporting CLWHIV including job-aids, technical guidance for HCWs, mobile applications and e-learning platforms for HCWs on the frontline.

KidzAlive is defined as a multicomponent child-centred capacity building model of care that facilitates the provision of integrated HIV care to CLWHIV aged 2–12 years (www.kidzalive.co.za). This model is a blend of several theories, which are Bandura’s Social Learning Theory [57], Piaget’s Cognitive Developmental Theory [58], Vygotsky’s Sociocultural Theory, and Play Therapy Theory [59–61].

### From development to scale-up

KidzAlive has now been endorsed by the South African national government and all provincial health departmentsas their differentiated HIV care model for all children aged 0–12 [62]. It forms part of existing National Guidelines including the National Adherence Guidelines and The National Disclosure Guidelines. In KwaZulu-Natal, Zoë-Life continues to support the Provincial Department of Health’s 95-95-95 strategy for children aged 0–19 years [62].

### Components of KidzAlive

KidzAlive comprises three key interventions:

- Intervention (capacity building)
- KidzAlive Talk tool storybook, and
- Child-friendly spaces.

### KidzAlive capacity building

#### KidzAlive HCW training

Zoë-Life is guided by district management teams to select HCWs responsible for providing HIV care to children in PHCs to be trained on KidzAlive [38]. Ideally, minimum of two HCWs, usually and nurse are selected. The trainers of this course are qualified nurses and social workers with extensive experience in training and working with children and adolescents in HIV management [38]. Zoë-Life then conducts a 5-day deductive classroom course which covers the following: child-rights, play therapy techniques, communicating with children, child-friendly spaces, stages of childhood development and dealing with children in distress [38].

#### KidzAlive mentorship: KidzAlive trained HCWs are mentored by their trainers post-training

The mentorship process takes place at the healthcare facility or community-based organisation (CBO) where KidzAlive trained HCWs usually provide HIV care to children. The KidzAlive mentor first demonstrates the procedure of providing care to a child-PCG pair in a process called “Preceptorship”. Following preceptorship, the HCWs are required to demonstrate their competence by successfully providing KidzAlive influenced HIV care to three sets of child-PCG pairs seeking any of the following: HIV testing, HIV disclosure, and adherence counselling. The mentor observes the process and grades the sessions using a mentorship checklist reflecting specific child-friendly skills mentees should master. The mentorship proficiency score is > 65%. If they score lower, the mentorship process is repeated [38, 56].

#### KidzAlive talk tool storybook (talk tool)

An overview of the Talk tool has already been given in the previous section. KidzAlive trained and mentored HCWs are provided with the Talk tool, a colourful cartoon-based job-aid used in the provision of HIV care including HIV counselling and testing, Disclosure counselling, Adherence Counselling and HIV patient literacy. When providing HIV care, the HCW places the storybook on a table. The side of the storybook with the narrative faces the HCW while the child sits opposite the HCW facing the side of the storybook with cartoon illustrations of the story being narrated [38, 56].

#### Child-friendly spaces

These are areas in healthcare facilities that are designated for providing HIV care to CLWHIV. Usually Zoë-Life provides the facility with a child-friendly space toolbox and some furniture that include tables, chairs or playmats depending on the available space. During KidzAlive training, HCW are trained to create child-friendly spaces and to utilise them during the process of providing HIV care to children. Children are encouraged to participate in the setting up, decoration and maintenance of the space. These spaces are expected to create a child-friendly ambience in the facility and to improve children’s experiences of care in poor resourced PHCs [38, 56].

## Methods

### Study design

Using a qualitative explorative, descriptive and contextual design, rooted in the interpretive paradigm [63], we explored the mechanism through which the KidzAlive Talk tool, a child-centred job-aid for HCWs facilitates child-participation during HIV consultations in PHC clinics implementing the KidzAlive model. To understand this mechanism, we also realised that we needed to explore the barriers preventing CLWHIV from being active participants during their care. In this study, HIV services included HIV counselling and testing (HCT), disclosure support, adherence support and general health education for children receiving HIV care at PHCs. The COREQ checklist [64] was used to ensure that the standards for reporting qualitative research were met.

### Study setting and participants

This study was part of a larger pragmatic quasi-experimental study. The study aim was to evaluate the impact of KidzAlive trained and mentored HCWs on the quality of HIV care provided to children aged 5–12 in 40 PHCs in KwaZulu-Natal Province over a period of 6 months (September 2018 to February 2019). In the parent study, 40 PHCs in four districts (eThekwini, uMkhanyakude, Zululand, and uMgungundlovu) were selected. These clinics were chosen because they were part of the KidzAlive scale-up sites where Zoë-Life was collaborating with the KwaZulu-Natal Provincial Department of Health. This scale-up process involved the following:

- KidzAlive training and mentorship of 80 HCWs (two from each of the 40 sites),
- Providing each HCW with the KidzAlive Talk tool,
- Providing each site with a child-friendly space toolbox for creating a child-friendly space.

This qualitative study builds on the quantitative results on the outcomes of KidzAlive capacity building (unpublished) by further exploring the mechanism through which the Talk tool improves child-participation during HIV consultations in PHCs implementing the KidzAlive model.

### Participant selection

#### Selection of HCWs

In the parent study, we purposively selected 80 HCWs who were nominated by PHC line managers to participate in the KidzAlive programme. These HCWs were assured of their freedom to withdraw from the research arm of the intervention at any time but not the training or mentorship itself, which was a service delivery activity central to patient care and was therefore compulsory for nominated HCWs. The distinction between the service delivery arm and research arm of the programme was clarified to the participants.

While we hoped to achieve data saturation, this was not the criteria for determining the sample size. We endeavoured to interview all the 80 KidzAlive trained HCWs spreading over 40 PHCs across four districts, participating in the parent study, but we finally interviewed only 20 HCWs due to funding constraints. The selected HCWs included eight (*n* = 8) nurses and 12 HIV/AIDS counsellors. These 20 HCWs were conveniently drawn from five PHC clinics due to their proximity to central towns/cities for easy access by the research team, and this was purely a decision based on financial considerations. We fully acknowledge this as an important limitation as it may have contributed to selection bias, thereby limiting the scientific rigour of our findings.

#### Selection of child-PCG pairs

We also conveniently selected 30 willing child-PCG pairs who had received HIV services from a KidzAlive trained and mentored HCW using the Talk tool. Notably, we only interviewed child-PCG pairs that had visited the healthcare facility on the day of data collection; hence we may have missed important perspectives from other child-PCG pairs. This approach was solely based on practicalities and its potential for introducing bias is duly acknowledged.

### Data generation procedures

#### Training of research assistants

Four research assistants (RAs) experienced in qualitative research methods were provided refresher training on qualitative research data generation processes. These RAs were fluent in the two languages (English and isiZulu) dominant in KZN Province. All interviews were audio-recorded (with participants’ assent and consent) and later transcribed verbatim by experienced transcribers before translation to English. The RAs also took field notes during each interview. Twenty per cent (20%) of the randomly identified transcripts were subjected to back-translation by the lead investigator (CM) for quality check. No major inaccuracies were identified during this quality check process. The interview questions explored participants’ perceptions of the barriers preventing their children from actively participating during care consultations. The focus was also on HCWs’ experiences of receiving HIV care with the aid of the KidzAlive Talk tool and how it had affected their engagement and participation in the care process.

### Field-testing of data collection tools

All data collection tools (interview guides) used in this study were pilot tested at 10 PHC facilities in King Cetshwayo District, KZN in July 2018. This district is one of the pilot districts where the comprehensive KidzAlive model was tested by Zoë-Life. Ten KidzAlive HCWs and 10 child-PCG pairs were interviewed from each of the 10 pilot facilities by a trained RA. During the pilot study, the interviewer first used an interview guide written in English, which was verbally translated to isiZulu during the interview process. These interveiw guides were developed to answer the key research questions of the parent study described in the “Study setting” section above. The authors synthesised data from participant responses related to the Talk tool and the barriers preventng child-participation during HIV care. After conducting two interviews with HCW participants, it became clear that the tools had to be translated to isiZulu before the interview as the RA struggled to translate some words from English to isiZulu during the interview. Finally, each question had both English and isiZulu versions as the participants used both languages interchangeably. The responses were also written in both isiZulu and English. Repetitive questions were removed while others were simplified to mitigate ambiguity. A copy of the key informant interveiw guides used in this study is available *(See* Additional file 1*).*

#### Interviews with children and PCGs

RAs interviewed 30 child-PCG pairs after they gave assent and consent to participate in the study. A child-friendly story-based information sheet was developed to improve children’s understanding of the research process and procedure. Thereafter, the child-PCG pairs were referred to the HIV counselling room with a child-friendly space where they received HIV care from a KidzAlive trained and mentored HCW using the Talk tool. After their HIV care consultation, the child-PCG pair were referred to another private room where they were each interviewed by an RA, separately. The average duration of each interview was 40 min. Again, it is important to emphasise that these interviews were conducted in the context of a bigger research study which used one tool, therefore, some questions related to the Talk tool were linked to others which are not part of this paper. The data collection tools for children and PCGs included questions around their perception of the Talk tool, their feelings towards the HCW during their session, and the HCW’s ability to allow them to participate in the care process. Further, the questions focussed on comparing the Talk tool session to previous sessions without the job-aid, and their perception of the characters and story and the cartoon illustrations in the storybook and their understanding of the stories that were read by their HCWs. PCGs were asked additional questions concerning their perception of the Talk tool-enhanced care process, their willingness to allow HCWs to engage with the child, reservations, concerns, length of the session and their child’s reaction to the Talk tool and enhanced care process. While the tool used to collect data may have been improved through individual inputs from the members of a study team (which constituted seven members), it was not subjected to formal inter-rater reliability testing.

#### Interviews with KidzAlive trained and mentored HCWs

KidzAlive trained and mentored HCWs were interviewed 6 months after the training and mentorship period (February 2019). These interviews were conducted at the PHC facility in the HCW’s consultation room or other free rooms at the facility, to avoid disruptions. Each in-depth interview lasted for about 1 hour. The interview questions probed HCWs’ perception of the tool, its illustrations, its role in the care process, its narration, metaphors introduced by storytelling, children and PCGs’ reaction to the Talk tool, its ability to influence the child’s care experience, experiences of children and their PCGs and many more.

### Data analysis

The data analysis process started with transcription and translation of the data from isiZulu to English, which was done by a qualified and experienced bilingual translator with knowledge of the KidzAlive intervention to ensure conceptual equivalence [62]. Coding of data was conducted by CM and KS. We adopted a thematic analysis method which followed Ritchie and Spencer’s five-stage data analysis framework [18] which followed the following simple steps:

- Familiarization
- Identifying a thematic framework
- Indexing
- Charting
- Mapping and interpretation

To answer the research question, we used a constant comparative and modified grounded theory analysis method which facilitated a critical comparison of *a)* inherent barriers to child-participation during HIV care consultations and *b)* mechanism of how the Talk tool had addressed these barriers and facilitated child participation in the HIV/AIDS care process.

### Measures to ensure the trustworthiness of the study

We used credibility, dependability, transferability, and confirmability [19] as the criteria for ensuring the trustworthiness of our findings. To ensure credibility, we adopted research methods that are well established in qualitative research. We also interviewed multiple data sources i.e., key stakeholders involved in KidzAlive including: KidzAlive trained HCWs and child-PCG pairs which facilitated corroboration of important information and triangulation of responses. Thick descriptions of the phenomenon under study presented in the background, methods and findings enhanced the transferability of the study findings. To ensure dependability and confirmability, we followed a peer reviewed protocol and documented the research steps taken from the start of a research project to the development and reporting of the findings.

### Ethics approval and consent to participate

Ethical approvals were received from the Directorate for Health Research and Knowledge Management at the KwaZulu-Natal Department of Health (KZ_201809_011) and the University of KwaZulu-Natal’s Biomedical Research Ethics Committee (BREC) (BE298/18). The KwaZulu-Natal Department of Health Provincial HIV, AIDS and STIs Directorate provided support letters which were presented to PHCs to gain access to each facility. Permission to use Zoë-Life’s KidzAlive programme for research purposes was sought from the Zoë-Life Directorate. Our RAs were trained on the informed consent/assent process. We sought written informed consent from all adult participants before conducting the respective interviews. We also sought written parental consent for children below the age of 18 years. The information in these sheets was verbally explained to children with the aid of two different sheets. RAs explicitly used age-appropriate language for each child. For children aged 5–7 years, a child-friendly story-based information sheet was used to increase their understanding for them to give informed assent. For older children aged 8–12 years, we used a more advanced information sheet, while maintaining toned-down language depending on the child’s ability to understand and this was at the RA’s discretion.

## Results

Of the 20 KidzAlive trained and mentored HCWs that participated in this study, 19 were females and one was male, twelve HCWs were HIV/AIDS counsellors and their age and work experiences varied widely. Most children were aged 7–8 years (*n* = 11) and 11–12 years (*n* = 9), respectively. Most of the children were female (*n* = 17). All the children were school-going and were on highly active antiretroviral therapy (HAART). Nearly half of the children (*n* = 14) were either fully (*n* = 9) or partially (*n* = 5) disclosed to (Table 1). Of those that were either partially or fully disclosed to, over two-thirds (*n* = 14) were disclosed to when they were aged between 6 and 8 years. Most PCGs were either married or cohabitating (*n* = 19) and at least half (*n* = 24) were aware of their HIV seropositive status (Table 1).

**Table 1:** Demographic characteristics of the children, PCGs and KidzAlive trained and mentored HCWs

Category of Participants	Variable		Frequency
KidzAlive trained and mentored HCWs	Sex	Male	1
		Female	19
	Age (in years)	18–25	3
		26–30	4
		31–40	8
		40+	5
	Professional designation	Professional nurse	8
		HIV/AIDS counsellor	12
	Experience working with children (in years)	1–3	9
		4–6	2
		7–9	7
		10+	2
Primary Caregivers (PCGs)	Sex	Male	3
		Female	27
	Age (in years)	18–25	8
		26–30	10
		31–40	9
		40+	3
	Marital status	Married/cohabiting	19
		Single	7
		Divorce/separated /widowed	4
	Relationship to the child	Biological mother	19
		Biological father	1
		Grandparent	2
		Uncle/Aunt	4
		Sibling	4
		Other	1
	Level of education	No formal education	2
		Up to primary school	3
		Up to high school and beyond	25
	HIV status	HIV positive	15
		HIV negative	9
		Unknown	6
Children	Age (years)	5–6	4
		7–8	11
		9–10	6
		11–12	9
	Sex	Male	13
		Female	17
	Age at diagnosis (in years)	0–2	12
		3–4	9
		5–6	3
		7–8	3
		9–10	2
		11–12	1
	Child on HAART	Yes	30
		No	0
	Child going to school	Yes	30
		No	0
	Disclosure status of child	Not disclosed	16
		Partially disclosed	5
		Full disclosure	9
	Child’s age at disclosure (in years)	2–5	1
		6–8	10
		9–12	3

The study sought to explore the mechanism through which the KidzAlive Talk tool, a child-centred job-aid for HCWs, facilitates child-participation during HIV consultations in PHCs implementing KidzAlive. To determine this mechanism, we also recognised the need to understand the barriers preventing CLWHIV from being active participants during their care. Therefore, we present our findings as follows:

- Themes related to the barriers to child-participation in the HIV care process were as follows:

1) PCG limiting the child’s involvement due to fear of traumatising them; 2) HCW’s limited knowledge and skills to deliver child-centred HIV care; 3) Childhood developmental stage-related limitations; and 4) Healthcare institutional paternalism.

b) Themes related to understanding the mechanism through which the Talk tool addresses the barriers to child participation were as follows:

1) Use of simple language and terminology to cater for children at various stages of development; 2) Alleviation of HCW and PCGs’ fear of possible psychological harm to the child; 3) Use of storytelling and colourful cartoon illustrations for child edutainment; 4) Versatility of the Talk tool allowing for multiple utilities; and 5) Tackling institutional paternalism limiting child-involvement in process of care.

Because these themes were related, their results are presented concomitantly (where a barrier is linked with the mechanism which addresses it) below.

### Childhood developmental stage-related limitations Vs use of simple language and terminology to cater for children at various stages of development

The study identified childhood developmental stage-related limitation of children as an important barrier to child-participation. This barrier seemed to be addressed by using simple language and terminology which catered for children at various stages of development. Children stated that the Talk tool was written in, and illustrated using appropriate language, terminology, metaphors, and visuals appropriate for children of varying cognitive-developmental competencies (5–12 years). HCWs concurred with the children that these key attributes boosted their confidence to talk to children and their PCGs about difficult topics related to HIV (P1, P7, P9, P14-P20). Metaphors such as “a germ in your body” referring to HIV; “goodnight medicine” referring to ARVs “putting the germs to sleep” implying that HIV cannot be cured but ART can suppress it, was praised by both HCWs and child-PCG pairs for making the unfamiliar more understandable and manageable for children. One PCG shared the following.

*“The HCW now uses quite simple language to explain to my child about HIV, and we understand it. I especially appreciate this tool that she uses, of telling the story about Sibusiso. It alleviates my fear and those of my child. I know no harm will befall my child”* (PCG).

HCWs and PCGs stated that the fear of traumatising the child by discussing HIV was the main factor preventing them from actively engaging with children during HIV consultations (H2, H3, H7, H14, H19, H20). HCWs commended the “Feeling faces” tool for being an innovative tool for alleviating their fears by enabling them to constantly check the child’s emotional state throughout the process of care to prevent trauma and ultimately confusing the child (H2, H7, H14, H20). Some of the participants shared the following.

*“We want to talk to children to ensure that they are not scared and that they feel at home at the health facility. Sometimes, when you are providing a service, you forget to check how the child is feeling about the on-going conversation. The Talk tool allows us to ask the child some questions about how they are feeling [Feeling faces tool], using pictures and thereby providing valuable feedback to the HCW”* (HIV counsellor).

*“Dealing with sick children is always a big challenge as they are often scared and overwhelmed by the apprehension of medical procedures, and as HCWs, we are also emotionally affected by this uneasiness. However, the colourful illustrations, stories from this book and the “Feeling faces tool” now make it easier to explain to children without harming them emotionally, which I suppose reassures us and we feel less apprehensive”* (Nurse).

The translation of the Talk tool to four South African languages (Sotho, isiZulu, Afrikaans, and English) was another innovation which was appreciated by most HCWs.

Some PCGs aired their concerns regarding the use of the Talk tool only at PHCs. They recommended that the Talk tool should have an accompanying comic book for children to be given as take-home support. They added that this comic book would help them remember the information taught at the healthcare facility (P1, P2, P4, P20). For example, one participant said.

*“The Talk tool should have a small booklet to give to children to read at home, like a comic book so that they don’t forget the information taught and they can refer back to the book if they want to increase their knowledge about their illness”* (PCG).

### PCG limiting the child’s involvement due to fear of traumatising them Vs alleviation of HCW and PCG’s fear of possible psychological harm to the child

Most PCGs commended the “Primary Caregiver Preparation” section at the beginning of the Talk tool storybook, which provides the PCG of a child with information on the journey that they were embarking on with their child (P1, P3, P4-P13, P20). All the PCGs reported that the process provided them with psychosocial support to overcome their fears and to prioritise their children’s needs above theirs and increased their willingness to encourage their children to actively participate in the process of care (P1-P30). One of the participants explained.

*“The HCW also uses the storybook to prepare me as the caregiver of the child to be aware of the journey I am about to embark on, together with my child. I think this psychosocial process prepares you to deal with both good or bad news and how to navigate the care process if the child is found to be HIV positive. This process encourages us to partner with the HCW in looking after the child and ensures that they receive the best care possible, here at the hospital and that they are supported at home as well”* (PCG).

One of the barriers to limited engagement of CLWHIV is usually the unwillingness of PCGs who are usually fearful of people finding out that their children are on ART, which would also imply that they are also living with HIV. One PCG cited that these fears were driven by stigma, which is still rampant in many South African communities. By limiting their children’s knowledge of their condition and their interaction with the HCW during care consultations, one PCG cited that it made them feel protected from stigma and judgement by family members, friends, and the community at large.

PCGs welcomed the use of the “Hand of Safety Tool”, which encourages the child to identify people that are in the child-PCG pair’s circle of support. During this process, the HCW describes “Inside stories” i.e., stories that can only be told to people in the circle of support listed on the hand of safety and “Outside stories”, which should only be told to other people. Using this tool, children are encouraged by the KidzAlive trained and mentored HCW to only disclose their status or discuss their problems about their condition with only members of the circle of support to avoid inadvertent disclosure to other people. All the PCGs indicated that this tool had alleviated their fear of the child disclosing their own HIV positive status to other people and had increased their willingness for their children to be more engaged and involved in their care process (P1-P30). One of the participants explained.

*“The nurse showed my child the people who are in their hand of safety. I like that because it ensures that the child keeps secrets, which protects us from being stigmatised by family and community members...”* (PCG).

One of the key barriers mentioned by both HCWs and PCGs is the HCW’s limited knowledge and skills to deliver child-centred HIV care. Most HCWs indicated that they had not received training before the advent of KidzAlive and therefore, they were fearful of inadvertently disclosing to CLWHIV who are not aware of their HIV serostatus. All the HCWs appreciated the Talk tool, citing that the simplified HIV jargon, coupled with a simplified HIV care process gave them the confidence to start conversations and engage children under their care in meaningful conversations that they too could understand.

### Childhood developmental stage-related limitations Vs use of storytelling and colourful cartoon illustrations for child edutainment

Childhood developmental stage-related limitations were noted as barriers to child-participation as PCGs felt that their children were not mature enough to understand and process the information regarding their condition. As a way of engaging children, storytelling, and colourful cartoon illustrations were regarded as acceptable innovations for tackling these age-related barriers by all participants. Based on our observations during site visits, the children were overly excited during the care process. They wanted to continue discussing the story of *‘Sibusiso the Frog’*. All the children indicated that they had enjoyed the story, especially the illustrations of *Sibusiso the Frog*, his grandfather, the nurse, and all his friends. Older children mentioned that the story used simple language and provided clear information that they could understand with relative ease. Importantly, they indicated that the story was entertaining (C1-C20). All the children reported that storytelling had alleviated the anxiety caused by routine clinical procedures and created a better understanding of why these procedures had to be conducted (C1-C30). Some of the children shared the following:

*“I want to be brave like Sibusiso, so I won’t cry when the nurse pricks my finger. It has to be done so that we can check if my soldier cells are fighting off the germs in my body”* (Child).

*“The nurse told me about the importance of taking ‘Goodnight medicine to keep the germ asleep’. She told me that I must take this medicine every day just like Sibusiso so that my soldier cells can fight bad germs”* (Child).

In their narratives, PCGs highlighted that the Talk tool had empowered them to reinforce medical advice given by the KidzAlive trained and mentored HCWs through storytelling techniques when communicating with their children (P1, P7, P13-P25, P27-P30). One participant said:

*“We have also learnt some useful words to use with our children and we now use the story of Sibusiso to reinforce medical advice and to disclose to the child...”* (PCG).

### HCWs' limited knowledge and skills to deliver child-centred HIV care Vs versatility of the talk tool allowing for multiple utilities

The key barrier to child engagement related to limited knowledge and skills to deliver child-centred HIV care by HCWs seems to have been addressed by the Talk tool. HCWs cited that the Talk tool served as a counselling guide, educational resource, storybook for entertaining children, and a job-aid aligned with HIV testing, HIV counselling and the Disclosure Guidelines currently used in South Africa (H1-H20). The Talk tool was particularly applauded by HCWs for being structured according to the HIV cascade from HIV testing to adherence support, and wellness (H1-H20). However, one PCG cited that the engagement of CLWHIV should continue from the PHC to their homes to facilitate continuity of care at home. To that effect, several PCGs requested a tool to be packaged in a booklet that children can take home to reinforce key messages and facilitate continuity of care (P1, P2, P9, P20-P25, P30). Participants shared their views as exemplified below:

*“The Talk tool is structured following HIV testing, ART, disclosure, and adherence guidelines. Therefore, it provides HCWs with simplified and flexible guidance to provide accurate information. It is also structured in a gradual and progressive process, which makes it easy to use for all the stages of care from pre-test counselling of PCGs, pre-test counselling of children, to pricking the child and giving out results etc”* (Nurse).

*“The Talk tool is available in multiple South African languages, which makes it easy to use. However, they should develop a small booklet to give to children”* (PCG).

*“The Talk tool is not just a job-aid for us, but it is also a storybook for children. Its structure means that it can be used by both the HCW and the child, simultaneously. There are no other tools that I have come across that have these multiple benefits”* (Nurse).

HCWs especially HIV counsellors also reported that use of the Talk tool as a job-aid greatly minimised their dependence on memory or specialised support from their senior supervisors (H1-H20). One of the participants shared the following.

*We now have a job-aid that reminds us of the guidelines and gives a practical way of providing HIV services for children that are aligned to the guidelines. The Talk tool is combining HIV testing guidelines, disclosure guidelines and adherence guidelines into a remarkably simple format, which is easy for us to follow”* (Nurse).

HCWs reported that the Talk tool was easy to use and follow even in the absence of KidzAlive training or mentorship (H2, H3, H9). The following was stated.

*“Even with no training or mentorship, the Talk tool can guide you, and enable you to easily provide child-friendly services because it is easy to follow and understand. You can even give it to a non-KidzAlive trained HCW and they can effectively use it”* (Nurse).

### Healthcare institutional paternalism Vs tackling institutional paternalism limiting child-involvement in process of care

One of the HCWs identified institutional paternalism, which alienates CLWHIV from their care with PCGs rather than children being in the centre of the care process and participating in decision-making regarding their HIV care. She also cited that while the process of counselling and testing seemed quite simple for adults, it was complex for children due to HCW and PCG paternalism, which results in children playing a passive role, resulting in the underestimation of their capacity, opinions, questions and wishes. The Talk tool seems to break this deeply seated paternalism by introducing a novel way of providing HIV care to CLWHIV, which makes their involvement in the process of care mandatory by mainstreaming the use of the Talk tool in every step of the HIV care process. As such, HCWs and PCGs have no choice but to actively involve the children during HIV care consultations.

## Discussion

The findings of this study suggest that the Talk tool has been instrumental in addressing the barriers to child-participation during HIV care. These barriers included: PCG’s limiting the child’s involvement; HCW’s limited capacity to deliver child-centred HIV care; Childhood developmental stage-related limitations; Healthcare institutional paternalism.

The mechanism through which the Talk tool addresses the above barriers and facilitates child-participation during HIV care consultations include: Use of simple language and terminology to cater for children at various stages of development; Alleviation of HCW and PCG’s fear of possible psychological harm to the child; Use of storytelling and colourful cartoon illustrations for child edutainment; Addressing institutional paternalism related to child-involvement in process of care and the versatility of the Talk tool. Based on these findings, the Talk tool partly meets the criteria of the five levels of child-participation in healthcare proposed by Harry Shier [16] as it allows children to be listened to, and are facilitated in expressing their views and that their views are considered.

The mainstay of the Talk tool in facilitating child-participation during HIV consultations is that it uses simple language and terminology to cater for children at various stages of development. This is consistent with the literature on health education and promotion where metaphors are used to explain and clarify scientific or complex medical phenomenon to improve understanding and facilitate behaviour change such as adherence to medication [65]. Several studies suggest that one of the barriers to adherence to medication or advice from healthcare providers is the inability to communicate scientific medical jargon or articulate physiological processes using simple, understandable, and non-intimidating language [3, 39, 44, 65].

Furthermore, the metaphors used in the storybook do not specifically name the disease or complex mechanisms around how HIV affects their body to ensure that children receive information that is consistent with both their developmental stage and disclosure status. These findings are consistent with those reported in Namibia, where a comparable child-friendly job-aid called the “Disclosure Storybook” was used to support status disclosure for children aged 7–12 years [39, 66]. Like the KidzAlive Talk tool, this Namibian Disclosure Storybook job-aid used metaphors, such as the reference to medication as “a germ in your body” and scientific jargon such as CD4 cells being referred to as “Soldiers that fight germs”. These metaphors make it easier for HCWs to explain to children and their PCGs important information around HIV infection, how ART works in their bodies and the importance of adherence to treatment [39, 44].

The Talk tool provided the HCW with simplified guidelines to share age-appropriate and accurate information with children. The storybook provided KidzAlive trained and mentored HCWs with a stepwise guide to follow when providing HIV services along the HIV care cascade from provider-initiated counselling and testing (PICT), initiation of ART, adherence to ART, viral load monitoring to disclosure. The study highlighted that its simplicity and user-friendliness minimised the amount of support and supervision of PHC staff using it, especially HIV counsellors, thereby saving the much-needed resources and time. These findings resonate with literature describing the value of job-aids to frontline HCWs and their function as content reminders by assisting HCWs to remember key information [42, 47, 67].

One of the main barriers to engagement and participation of CLWHIV is that their parents deter children’s involvement. There are many reasons why this is the case, but the root cause seems to be related to the perceived stigma and fear of possible psychological harm to the child. One key issue that emerged as the main barrier was that of inadvertent disclosure to other people. The findings of this study provided important insights into how the Talk tool uses the hand of safety tool to prevent inadvertent disclosure from taking place. We can therefore safely conclude that the Talk tool facilitates child-participation by reassuring the PCGs that their secret (their HIV positive status or child’s HIV status) is safe, thereby providing an incentive for them to willingly allow HCWs to freely engage with children. This finding is unique to this study as we could not identify any literature where a job-aid incentivised CLWHIV’s participation in their care programmes.

The versatility of the KidzAlive Talk tool makes it flexible for use as an engagement tool, which encourages child-participation as it functions as a counselling guide, educational resource, storybook for entertaining children, and a job-aid aligned with HIV testing, HIV counselling and the Disclosure Guidelines currently used in South Africa. These factors make it a cost-effective solution for improving the quality of HIV care for children in poor resourced PHCs. Such settings require low cost, high impact healthcare solutions with the potential to reach many CLWHIV in desperate need of quality HIV care [13]. It may also be considered a useful alternative to formal training of HCWs on child-centred care in poor resourced settings.

There were sound suggestions by PCGs of expanding the Talk tool to a comic book, which children could take away to allow for the continuation of care at home and reinforcement of HIV care information. Comic books have been successfully used as a viable HIV/AIDS edutainment approach for children and young people [68]. These comic books are guided by behaviour change theories, which are premised on the notion that before individuals reduce their levels of risk or change their behaviours, they need to first understand the basic facts about HIV and AIDS, adopt positive attitudes, acquire a set of skills, and have access to appropriate services [68]. By receiving a comic book based on the characters of the Talk tool, CLWHIV can potentially learn about their condition and protective behaviours necessary for them to prolong their lives and protect their loved ones from contracting the virus from them now and in future.

Paternalist attitudes of PCGs and HCWs were considered a significant barrier to the participation of CLWHIV in their care. The Talk tool seems to have effectively addressed this barrier by making it compulsory for HCWs to use the tool with every child receiving HIV care along the HIV-care cascade. As a result, CLWHIV are no longer passive recipients of care, at least in those PHCs implementing the KidzAlive model.

However, it is important to note that the barriers to participation by CLWHIV highlighted in this study related to PCG and HCW’s fear of emotional harm to CLWHIV are valid and are widely reported [3]. However, considering that HIV is now considered as a chronic condition, which is communicable, children should be aware of their condition and how to protect their families, friends, and future sexual partners. Therefore, child-participation supersedes that fear in the interest of public health and safety. A critical review of the perceptions of children’s participation in their healthcare [69] advocated for increased involvement of children in the care process and decision-making process [70]. In this aforementioned review [69], children expressed feelings of ownership and empowerment when involved in their healthcare. However, there were challenges with ascertaining the level of involvement and participation of children in the care process. The uncertainties included key issues raised by PCGs in this study, such as the developmental stage limitations of children, which may affect their full understanding of their condition, and the possibility of trauma from receiving bad news. These were among the challenges identified by other scholars [3, 71–73].

### Strengths and limitations of the study

Although we recruited participants from various facilities across four districts, our findings do not reflect the diversity of all the Talk tool users in KwaZulu-Natal Province and the rest of South Africa. This limits the generalizability of our findings. It therefore follows that the study does not provide objective measures of HIV outcomes such as HIV testing rates, disclosure rates and viral load results. However, this qualitative study provides future researchers with a starting point for rigorous studies that can measure the impact of the Talk tool on CLWHIV’s outcomes. It provides the impetus to design more rigorous and controlled studies, whose results can be generalised to the rest of South Africa and similar resource-constrained settings.

### Implications of the study

Understanding how and why existing interventions work is critical to replicating and adapting them, especially in low-resource settings with a high HIV prevalence among children. The Talk tool is an example of a promising cost-effective capacity-building intervention that can be used to improve the quality of care for children and health outcomes among CLWHIV and receiving care in PHCs in resource-constrained settings.

## Conclusion

This study provided evidence on the mechanism of how the Talk tool storybook addresses the barriers to child-participation and facilitates their participation in the HIV care process. Its use of child-friendly language, storytelling, colourful cartoons, and visuals, coupled with versatility as a storybook, job-aid, and guideline, make it a high impact tool for promoting quality care in PHC settings. However, more robust research still needs to be conducted to determine the impact of this innovation on children’s HIV outcomes. Nonetheless, the evidence generated from this study is compelling enough to recommend the scale-up of this innovation in low-resource settings.

## Supplementary Information

**Additional file 1.** Key Informant Interview Guides. This file contains the Key informant interview guides for healthcare workers, children and primary caregivers used to guide the interviews for data collection.

## Data Availability

Qualitative interview transcripts and the corresponding NVivo files are only visible to the direct research team and are not publicly available. However, data will be made available upon reasonable request to the corresponding author.

## Abbreviations

CLWHIV: Children living with HIV
PLHIV: People living with HIV
PCGs: Primary Caregivers
HCWs: Healthcare Workers
PHCs: Primary Healthcare Clinics
RA: Research Assistants

## References

[1] Kilkelly U, Donnelly M (2014). The Child’s right to be heard in the healthcare setting: perspectives of children, parents and health professionals the National Children’s strategy research series Office of the Minister for children Dublin.

[2] Donnelly M, Kilkelly U (2011). Child-friendly healthcare: delivering on the right to be heard. Med Law Rev.

[3] Mutambo C, Hlongwana K (2019). Healthcare workers’ perspectives on the barriers to providing HIV services to children in sub-Saharan Africa. AIDS Res Treat.

[4] Fairbrother H, Curtis P, Goyder E (2016). Making health information meaningful: Children's health literacy practices. SSM - Popul Health.

[5] Velardo S, Drummond M (2016). Emphasizing the child in child health literacy research. J Child Health Care.

[6] Bröder J, Okan O, Bollweg TM, Bauer U. Health literacy in children– towards a child-centered conceptual understanding: Janine Broeder. Eur J Pub Health. 2018;28(Suppl. 4):218–9.

[7] Abraham O, Wytiaz RM, Feathers AM (2019). Paediatric use of medications and adherence apps: a qualitative analysis of the perspectives of children and parents. J Pharm Pract Res.

[8] WHO (2010). Antiretroviral therapy for HIV infection in infants and children: towards universal access.

[9] Kim MH (2017). High self-reported non-adherence to antiretroviral therapy amongst adolescents living with HIV in Malawi: barriers and associated factors. J Int AIDS Soc.

[10] Kunapareddy CJ (2014). A qualitative assessment of barriers to antiretroviral therapy adherence among adolescents in Western Kenya. J HIV/AIDS Soc Serv.

[11] Bateganya MH (2015). Impact of support groups for people living with HIV on clinical outcomes: a systematic review of the literature. J Acquir Immune Defic Syndr.

[12] Lubombo M, Dyll LE (2018). A dialectic analysis of views on participation in HIV/AIDS communication of selected south African people living with HIV/AIDS: beyond the greater involvement of people living with HIV/AIDS. Critical Arts.

[13] Mutambo C, Shumba K, Hlongwana KW (2019). Child-Centred care in HIV service provision for children in resource constrained settings: a narrative review of literature. AIDS Res Treat.

[14] Organization, W.H (2017). Treat all: policy adoption and implementation status in countries: fact sheet.

[15] Vahdat S, Hamzehgardeshi L, Hessam S, Hamzehgardeshi Z. Patient involvement in health care decision making: a review. Iran Red Crescent Med J. 2014;16(1):e12454. 10.5812/ircmj.12454. 10.5812/ircmj.12454 PMC3964421 24719703

[16] Shier H (2001). Pathways to participation: openings, opportunities and obligations. Child Soc.

[17] Gyamfi E (2015). Benefits of disclosure of HIV status to infected children and adolescents: perceptions of caregivers and health care providers. J Assoc Nurses AIDS Care.

[18] Feinstein L (2010). Effect of disclosure of hiv status to children receiving art on six-month virologic suppression. *American journal of epidemiology*.

[19] Madiba S (2012). Patterns of HIV diagnosis disclosure to infected children and family members: data from a paediatric antiretroviral program in South Africa. World J AIDS.

[20] Yeap AD (2010). Factors influencing uptake of HIV care and treatment among children in South Africa - a qualitative study of caregivers and clinic staff. AIDS Care.

[21] Wright S (2017). Talking to children about their HIV status: a review of available resources, tools, and models for improving and promoting pediatric disclosure. AIDS Care.

[22] Demmer C. Experiences of families caring for an HIV-infected child in KwaZulu-Natal, South Africa: an exploratory study. AIDS Care. 2011;23(7):873–9. 10.1080/09540121.2010.542123. 10.1080/09540121.2010.542123 PMC3125458 21400305

[23] Health, S.A.N.D.o (2016). Disclosure guidelines for children and adolescents in the context of HIV, TB and non-communicable diseases.

[24] Organization, W.H (2011). Guideline on HIV disclosure counselling for children up to 12 years of age.

[25] Gyamfi E (2017). Prevalence of, and barriers to the disclosure of HIV status to infected children and adolescents in a district of Ghana. BMC Int Health Hum Rights.

[26] Vreeman RC (2013). Disclosure of HIV status to children in resource-limited settings: a systematic review. J Int AIDS Soc.

[27] Ubesie AC (2016). HIV status disclosure rate and reasons for non-disclosure among infected children and adolescents in Enugu, Southeast Nigeria. SAHARA-J: J Soc Aspects HIV/AIDS.

[28] Madiba S, Mokgatle M (2017). Fear of stigma, beliefs, and knowledge about HIV are barriers to early access to HIV testing and disclosure for perinatally infected children and adolescents in rural communities in South Africa. S Afr Fam Pract.

[29] Britto C (2016). Prevalence and correlates of HIV disclosure among children and adolescents in low- and middle-income countries: a systematic review. J Dev Behavioral Pediatr.

[30] Kiwanuka J, Mulogo E, Haberer JE (2014). Caregiver perceptions and motivation for disclosing or concealing the diagnosis of HIV infection to children receiving HIV care in Mbarara, Uganda: a qualitative study. PLoS One.

[31] Ford K (2018). Child Centred care: challenging assumptions and repositioning children and young people. J Pediatr Nurs.

[32] Taub S (2003). Learning to decide: involving children in their health care decisions. AMA J Ethics.

[33] Kilkelly U, Savage E (2013). Child-friendly healthcare.

[34] Myer L, Moodley K, Hendricks F, Cotton M. Healthcare providers' perspectives on discussing HIV status with infected children. J Trop Pediatr. 2006;52(4):293–5. 10.1093/tropej/fml004. 10.1093/tropej/fml004 16533799

[35] Kuo C (2019). Resilience and psychosocial outcomes among south African adolescents affected by HIV. Aids.

[36] Mahloko JM, Madiba S. Disclosing HIV diagnosis to children in Odi district, South Africa: reasons for disclosure and non-disclosure. Afr J Prim Health Care Fam Med. 2012;4(1):345. 10.4102/phcfm.v4i1.345.

[37] Naidoo G, McKerrow N. Current practices around HIV disclosure to children on highly active antiretroviral therapy. S Afr J Child Health. 2015;9(3):85–8. 10.7196/SAJCH.7957.

[38] Mutambo C, Shumba K, Hlongwana KW (2020). Post-training and mentorship experiences of KidzAlive-trained healthcare workers at primary healthcare facilities in KwaZulu-Natal, South Africa. Afr J Prim Health Care Fam Med.

[39] Brandt L (2015). Growing-up just like everyone else: key components of a successful pediatric HIV disclosure intervention in Namibia. Aids.

[40] Florez-Arango JF (2011). Performance factors of mobile rich media job aids for community health workers. J Am Med Inform Assoc.

[41] Okeyo ILA, Dowse R (2018). An illustrated booklet for reinforcing community health worker knowledge of tuberculosis and facilitating patient counselling %. J African J Primary Health Care Fam Med.

[42] Oka M (2019). Effects of a job aid-supported intervention during antenatal care visit in rural Tanzania. Int J Africa Nurs Sci.

[43] Reid-Searl K (2017). Puppets in an acute paediatric unit: Nurse’s experiences. Collegian.

[44] O'Malley G (2015). "if I take my medicine, I will be strong: " evaluation of a pediatric HIV disclosure intervention in Namibia. J Acquir Immune Defic Syndr.

[45] da Silva RDM (2017). Therapeutic play to prepare children for invasive procedures: a systematic review. J Pediatr.

[46] Jennings L (2010). Antenatal counseling in maternal and newborn care: use of job aids to improve health worker performance and maternal understanding in Benin. BMC Pregnancy Childbirth.

[47] Harvey SA (2008). Improving community health worker use of malaria rapid diagnostic tests in Zambia: package instructions, job aid and job aid-plus-training. Malar J.

[48] Beyers L, Phipps WD, Vorster C (2017). An introduction to teddy bear therapy: a systems family therapy approach to child psychotherapy. J Fam Psychother.

[49] Costantino G, Malgady RG (1994). Storytelling through pictures: culturally sensitive psychotherapy for Hispanic children and adolescents. J Clin Child Psychol.

[50] Carlson R, Arthur N. Play therapy and the therapeutic use of story. Can J Couns Psychother. 2007;33(3). https://cjc-rcc.ucalgary.ca/article/view/58625.

[51] Haigh C, Hardy P (2011). Tell me a story--a conceptual exploration of storytelling in healthcare education. Nurse Educ Today.

[52] Rossiter M, Garcia PA (2010). Digital storytelling: a new player on the narrative field. New Dir Adult Cont Educ.

[53] Day V (2009). Promoting health literacy through storytelling. OJIN: Online J Issues Nurs.

[54] Tevendale is third-year, F (2015). Using patient storytelling in nurse education. Education.

[55] Zoe-life (2010). KidzAlive talk tool job-aid.

[56] Mutambo C, Shumba K, Hlongwana KW (2020). User-provider experiences of the implementation of KidzAlive-driven child-friendly spaces in KwaZulu-Natal, South Africa. BMC Public Health.

[57] Bandura A (1977). Social learning theory.

[58] Piaget J. *Piaget’s theory*. In: Inhelder B., Chipman H.H., Zwingmann C. (eds) *Piaget and his school*. Springer Study Edition. Berlin: Springer; 1976. 10.1007/978-3-642-46323-5_2.

[59] Bratton SC (2005). The efficacy of play therapy with children: a meta-analytic review of treatment outcomes. Prof Psychol Res Pract.

[60] Landreth G (2002). Play therapy: the art of the relationship second edition.

[61] Schultz W (2016). Child-centered play therapy. Reason Papers.

[62] Unit, Z.-l.-K (2019). Scaling up of KidzAlive.

[63] Parker I (2013). Discourse analysis: dimensions of critique in psychology. Qual Res Psychol.

[64] Tong A, Sainsbury P, Craig J (2007). Consolidated criteria for reporting qualitative research (COREQ): a 32-item checklist for interviews and focus groups. Int J Qual Health Care.

[65] Lambert V, Glacken M, McCarron M (2013). Meeting the information needs of children in hospital. J Child Health Care.

[66] Beima-Sofie KM (2017). Pediatric HIV disclosure intervention improves knowledge and clinical outcomes in HIV-infected children in Namibia. J Acquir Immune Defic Syndr.

[67] Knebel E (2000). The use of manual job aids by health care providers: what do we know?.

[68] Obare F (2013). Effectiveness of using comic books to communicate HIV and AIDS messages to in-school youth: insights from a pilot intervention study in Nairobi, Kenya. Afr Popul Stud.

[69] Davies A, Randall D (2015). Perceptions of Children's participation in their healthcare: a critical review. Issues Compr Pediatr Nurs.

[70] Davies J, Wright J (2008). Children's voices: a review of the literature pertinent to looked-after children's views of mental health services. Child Adolesc Mental Health.

[71] WHO (2011). Guideline on HIV disclosure counseling for children up to 12 years.

[72] Hayfron-Benjamin A (2018). HIV diagnosis disclosure to infected children and adolescents; challenges of family caregivers in the central region of Ghana. BMC Pediatr.

[73] Mehta K (2016). Perspectives on disclosure among children living with HIV in India. Child Youth Serv Rev.

