# Supplementary material for: Exploring the mechanism through which a child-friendly storybook addresses barriers to child-participation during HIV care in primary healthcare settings in KwaZulu-Natal, South Africa
Source: BMC Public Health. 2021 Mar 16;21:508. doi: 10.1186/s12889-021-10483-8 (PMC7962374; doi:10.1186/s12889-021-10483-8)
Supplement: Supplementary file 1 — Additional file 1. Key Informant Interview Guides. This file contains the Key informant interview guides for healthcare workers, children and primary caregivers used to guide the interviews for data collection. [file 12889_2021_10483_MOESM1_ESM.docx]

***Key informant interview guides***

**Children and PCGs**

**These interview guides should be administered after providing a child with HIV services at a health facility in a private room**

| Questionnaire Code of PCG pair |  |
| --- | --- |
| Name of Facility |  |
| District Name |  |
| Name of interviewer |  |
| Date of Interview |  |
| Time of interview |  |
| Checked by |  |
| Verified by |  |

**Please answer the following questions after your session with the healthcare worker.**

**Demographic Data**

| **Primary caregiver information** | | |
| --- | --- | --- |
| Sex |  | Male |
|  |  | Female |
| Age of the PCG (years) |  | 18-25 |
|  |  | 26-30 |
|  |  | 31-40 |
|  |  | 40+ |
| Marital status |  | Married/cohabiting |
|  |  | Single |
|  |  | Divorce/separated /widowed |
| Relationship to the child |  | Biological mother |
|  |  | Biological father |
|  |  | Grand parent |
|  |  | Uncle/Aunt |
|  |  | Sibling |
|  |  | Other |
| PCG’s level of education |  | No formal education |
|  |  | Up to primary school |
|  |  | Up to high school and beyond |
| PCG’s HIV status |  | HIV positive |
|  |  | HIV negative |
|  |  | Unknown |
| **Child information** | | |
| Child’s age (years) |  | 5-6 |
|  |  | 7-8 |
|  |  | 9-10 |
|  |  | 11-12 |
| Child’s sex |  | Male |
|  |  | Female |
| Child’s age of diagnosis  (years) |  | 0-2 |
|  |  | 3-4 |
|  |  | 5-6 |
|  |  | 7-8 |
|  |  | 9-10 |
|  |  | 11-12 |
| Child on HAART |  | Yes |
|  |  | No |
| Child going to school |  | Yes |
|  |  | No |
| Disclosure status of child |  | Not disclosed |
|  |  | Partially disclosed |
|  |  | Full disclosure |
| Child’s age at disclosure (years) |  | 2-5 |
|  |  | 6-8 |
|  |  | 9-12 |

**Interview guide for Child**

| 1. Please tell me your experience when you arrived at the facility? Did the HCWs speak to you? 2. Did you enjoy your conversation with the HCW? 3. What did the HCW use when they were talking to you? 4. Did you enjoy the stories that you were told by the HCW? What were the stories about? 5. Did You enjoy the pictures? 6. Did the healthcare worker allow you to ask questions? 7. Did you ask any questions? 8. Did the Healthcare worker answer any of your questions? 9. Were there any differences in this visit compared to your initial clinic visits. If so, How so? 10. Did you understand the information that was shared by the HCW using the Talktool? 11. Did you get a chance to use the child-friendly space? 12. Did you understand what the space is for? Would you go back to the space next time you come back to the health facility? 13. Which activities did you do in the child-friendly space with the HCW? 14. Did you at any point feel left out in the process of care? If so, how so? |
| --- |

1. **Interview guide for primary caregiver**

| 1. What services did you receive when you came to the clinic? |
| --- |
| 1. Have you disclosed to your child? |
| 1. Was the Healthcare worker friendly to you? 2. Empathetic towards you? |
| 1. Did they provide you with adequate information regarding your needs? |
| 1. Was the HCW Empathetic towards your child? |
| 1. Did the HCW Spend enough time talking with your child to your satisfaction? |
| 1. Were you willing for the HCW to talk to your child? |
| 1. Do you feel that the HCW took your child seriously? |
| 1. Do you feel that the HCW encouraged and supported your child? 2. Do you feel that the HCW treated your child as an individual? |
| 1. Do you feel that the HCW used language understandable to your child? |
| 1. Do you feel that the HCW was aware of your child’s developmental status? |
| 1. Do you feel that the HCW provided your child with adequate information? |
| 1. Do you feel the HCW involved the child to your satisfaction when you were being provided the HIV services? |
| 1. Did the HCW use any new tool that you have never seen before? Do you think it improved your session? |
| 1. Did you like this talk tool which was used by the HCW? |
| 1. Do you think that your child liked the KidzAlive talk tool? |
| 1. Do you Think that the child-friendly space at the clinic improved healthcare experience for child? |
| 1. Do you think the child liked the activities done in the child-friendly Space? |
| 1. Were you comfortable with leaving your child to with the HCW talk to the Health Care worker in the child-friendly space? |
| 1. Do you think your child liked the Talk Tool? |
| 1. Do you think your child enjoyed the stories in the Talk tool? |
| 1. Do you think your child liked the animations in the Talk tool? |
| 1. Do you think the child understood the education provided by the Healthcare worker? |
| 1. Was your child engaging the whole time when they were in consultation? |
| 1. Was the session too long or too short? |
| 1. Do you think your child would like to go back to the same HCW again? |

1. **Healthcare Workers Exit Interview Guide**

| Sex |  | Male |
| --- | --- | --- |
|  |  | Female |
| Age (years) |  | 18-25 |
|  |  | 26-30 |
|  |  | 31-40 |
|  |  | 40+ |
| Professional designation |  | Professional nurse |
|  |  | HIV/AIDS counsellor |
| Experience working with children (years) |  | 1-3 |
|  |  | 4-6 |
|  |  | 7-9 |
|  |  | 10+ |

| **Perception of the Training and mentorship** | |
| --- | --- |
| 1 | Do you feel that the KidzAlive training, mentorship and job aids provided by LinkCARE (Zoe-Life), improved your confidence to provide HIV services to children? |
| 2 | Do you feel the mentorship you received from KidzAlive was sufficient for you to be able to effectively implement the programme? |
| 3 | Has the KidzAlive training, mentorship, and job aids (Talk Tool, Adherence Templates, Hand of Safety) increased your ability to interact (e.g playing, talking, listening, understanding) with the child? |
| 4 | Do you feel that KidzAlive training has improved the relationship between yourself (the healthcare worker) and the children you work with? |
| 5 | How has the KidzAlive training and mentorship program has influenced you to include the child when providing HIV Services to them instead of just the primary caregiver? |
| 6 | Do you feel that KidzAlive training improved your communication skills with children? |
| 7 | Do you feel that after you were trained on KidzAlive, you are now comfortable to talk to children from age 2 – 12 years about HIV? |
| **Key challenges** | |
| 8 | What is your perception of the Talk Tool? Has its helped you with your work when providing HIV care to children seeking HIV care? |
| 8 | Have you experienced any challenges with using the Talk Tool when providing HIV services to Children and their caregivers? |
| 9 | Have you experienced any challenges with accessing the job aids in the facility?(e.g. Sharing or Storage, Location) |
| 10 | Have you experienced any challenges with the willingness of Primary Caregivers to bring their children to be provided with HIV services? |
| 11 | Have you experienced any time constraint challenges when providing an HIV Service with the aid of the Talk Tool? |
| 12 | Are there any other challenges not mentioned above that you have faced when implementing the KidzAlive program? |
| 13 | If You have not faced challenges when providing children with HIV Services, what are the things you think enabled you to successfully implement with none of these challenges? |
| 14 | Do you think you have received adequate support from facility management (OM) in order to improve the implementation of the KidzAlive program? |
| 15 | Do you think you have received adequate support from other healthcare workers in the health facility to implement the kidzAlive Programme? |
| 16 | Do you think you received adequate additional technical support from the (Zoe-Life) after receiving training and mentorship? |
| 17 | Which HIV Services do you find to be the easiest to provide using the KidzAlive Talk Tool (Order from 1 to 3 with one being the easiest and 3 the least easy) HTS, Disclosure, Adherence Support? |
| 18 | Are you getting different responses from boys and girls regarding the visuals of the Talk Tool? |
| 19 | From your experience, have you found that the story of Sibusiso and his friends is appropriate for all age groups aged ranging from 2 to 12 years? |
| 20 | Which age groups are you finding challenges/difficulty with when using the Talk Tool (Age appropriateness)? |
| **Benefits of Child-friendly spaces in health facilities** | |
| 21 | Has the child-friendly space improved the quality of the HIV services that you provide to children? |
| 22 | Have you experienced any challenges with Creating a Child-friendly Space? |
| 23 | Have you experienced any challenges with the maintenance of the Child-friendly Space? |
| 24 | Are you using the child-friendly space in the clinic for children who access services? |
| 25 | Has the newly created child-friendly space at the clinic improved the quality of your service to children? |
| 26 | Has the child-friendly space improved your communication with the children whom you provide HIV services to? |
| 27 | Do you think that children benefit from a child-friendly space? |
| 28 | Do you feel that the child-friendly space is private enough for both children and caregivers? |
| 29 | Do you feel that children like the toys and games that are in your child-friendly space? |
| 30 | Do you feel the children like the location of the child-friendly space? |
| 31 | Do you feel that the Primary Caregivers of the children appreciate the purpose of the child-friendly space |
| 32 | Do you feel that the children you provide HIV services feel safe in the child-friendly space? |
| 33 | Do you think that children that have received the HIV services in the child-friendly space would like to come back to the child-friendly space? |
| 34 | Additional information which you would like the KidzAlive team to know to improve the programme? |
